# The Role of the Noradrenergic System in Eating Disorders: A Systematic Review

**DOI:** 10.3390/ijms222011086

**Published:** 2021-10-14

**Authors:** Jacopo Pruccoli, Antonia Parmeggiani, Duccio Maria Cordelli, Marcello Lanari

**Affiliations:** 1 IRCCS Istituto delle Scienze Neurologiche di Bologna, Centro Regionale per i Disturbi della Nutrizione e dell’Alimentazione in età Evolutiva, U.O. Neuropsichiatria dell’età Pediatrica, 40138 Bologna, Italy; antonia.parmeggiani@unibo.it (A.P.); ducciomaria.cordelli@unibo.it (D.M.C.); 2 Dipartimento di Scienze Mediche e Chirurgiche (DIMEC), Università di Bologna, 40138 Bologna, Italy; marcello.lanari@unibo.it; 3 IRCCS Azienda Ospedaliero-Universitaria di Bologna, Policlinico di S. Orsola, U.O. Pediatria d’urgenza, Pronto Soccorso Pediatrico e OBI, 40138 Bologna, Italy

**Keywords:** catecholamine, noradrenaline, norepinephrine, adrenaline, epinephrine, dopamine, eating disorders, anorexia nervosa, bulimia nervosa, binge-eating disorder, feeding behavior

## Abstract

Noradrenaline (NE) is a catecholamine acting as both a neurotransmitter and a hormone, with relevant effects in modulating feeding behavior and satiety. Several studies have assessed the relationship between the noradrenergic system and Eating Disorders (EDs). This systematic review aims to report the existing literature on the role of the noradrenergic system in the development and treatment of EDs. A total of 35 studies were included. Preclinical studies demonstrated an involvement of the noradrenergic pathways in binge-like behaviors. Genetic studies on polymorphisms in genes coding for NE transporters and regulating enzymes have shown conflicting evidence. Clinical studies have reported non-unanimous evidence for the existence of absolute alterations in plasma NE values in patients with Anorexia Nervosa (AN) and Bulimia Nervosa (BN). Pharmacological studies have documented the efficacy of noradrenaline-modulating therapies in the treatment of BN and Binge Eating Disorder (BED). Insufficient evidence was found concerning the noradrenergic-mediated genetics of BED and BN, and psychopharmacological treatments targeting the noradrenergic system in AN. According to these data, further studies are required to expand the existing knowledge on the noradrenergic system as a potential target for treatments of EDs.

## 1. Introduction

### 1.1. The Noradrenergic System in the CNS

The Central Nervous System (CNS) produces and utilizes a series of neurochemical transmitters (or neurotransmitters), binding to different receptors to stimulate intracellular signaling pathways. These molecules are synthesized by a limited number of neurons pertaining to specific brain nuclei, and project their extensive ramifications to widespread CNS regions, regulating signals form external and internal stimuli [1]. The catecholamines dopamine (DA), noradrenaline (NE), and adrenaline represent a relevant class among neurotransmitters. These are synthesized in the CNS, peripheral nervous system (PNS), in the adrenal medulla by the chromaffin cells, as well in the gastrointestinal tract in and in the kidneys [2,3].

**Figure 1:**
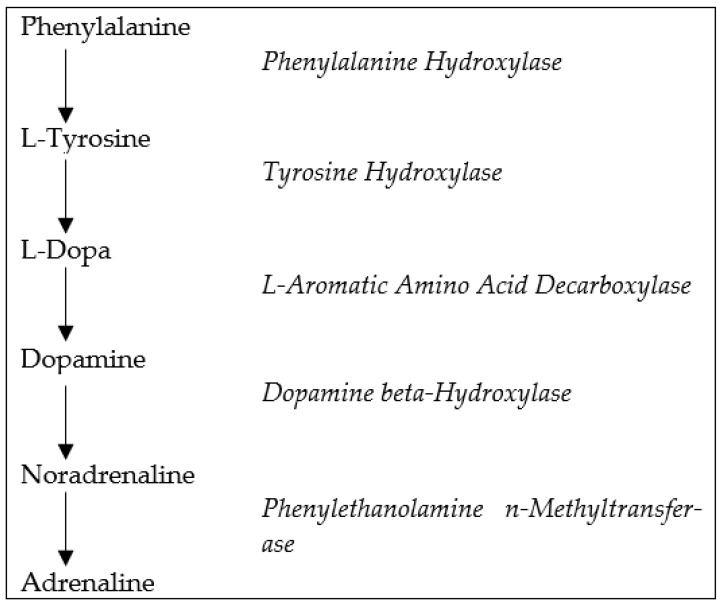
Pathways of the biosynthesis of catecholamines.

NE is synthesized in neurons, containing the dopamine-b-hydroxylase enzyme, catalyzing the conversion of DA to NE [4]. The main pathways for the synthesis of NE and other catecholamines are presented in Figure 1. Considering the specific anatomical disposition of the noradrenergic system, noradrenergic nuclei are located in the pons and medulla. The locus coeruleus (LC) represents the main noradrenergic nucleus, projecting its axons to a series of areas in the CNS [5]. Nonetheless, according to some researchers, noradrenergic neurons in the brainstem constitute a continuum of cells with blur boundaries and heterogeneous functions [5]. Ascending noradrenergic axons from brainstem neurons project diffusely to the neocortex, thalamus, hypothalamus, hippocampus, and virtually all of the CNS [6]. NE exerts its functions on three specific receptors coupled with G-proteins, named adrenoceptors b, a-1, and a-2 [7]. cAMP signaling is enhanced by b- and suppressed by a2-adrenoceptors via regulation of adenylyl cyclase, while a1-adrenoceptors activate phospholipase C signaling pathway [8]. Through the release of NE, noradrenergic neurons regulate a broad range of physiological and behavioral functions, such as arousal, memory, attention, appetite, and mood.

### 1.2. The Noradrenergic System and Feeding Behavior

Despite dopaminergic control of eating and feeding-related reward remaining the most extensively studied issue in this field, a series of relevant studies have investigated the noradrenergic regulation of feeding behavior. In a classic study of Grossman [9], the injection of exogenous NE into the lateral hypothalamus induced eating in rats, thus raising the hypothesis that endogenous NE could have a similar effect. Conversely, the introduction of NE into the perifornical hypothalamus has been shown to reduce feeding in rats [10], and NE depletion due to lesions of ascending ventral NE axons may lead to overeating, thus suggesting a role of NE in promoting satiety [11]. Further studies have investigated the specific organization of the noradrenergic regulation of feeding behavior. Together, the results of this research suggested an antagonistic organization of noradrenergic control of feeding, with a1-adrenoceptors activating descending inhibitory axons and suppressing feeding, and a2-adrenoceptors promoting nutrition by disinhibition of descending axons [6,12]. Relevantly, researchers have hypothesized that a1-adrenergic receptors may play a role in hypophagia. This hypothesis is supported by studies showing that a1-adrenergic receptors are present in the paraventricular nucleus (PVN) of the hypothalamus [13], an area of the brain associated with control of food intake [12].

Successively, researchers have investigated the potential effect of variations in the noradrenergic activity on feeding behavior. Desmethylimipramine, a noradrenalin uptake antagonist, has been injected into the prefornical hypothalamus of satiated rats, thus promoting eating behavior [14]. Sibutramine, a noradrenaline and serotonin reuptake inhibitor, has a documented anti-obesity effect on feeding in rats and humans; this effect, relevantly, may be reversed by an antagonist of a1-adrenoceptor such as prazosin [15]. Amphetamine-based medications, relevantly, have been frequently adopted in the treatment of binge eating disorder [16]. The inhibitory action of these drugs on eating behavior has been documented to be regulated by activation of brain NE and brain a1-adrenoceptors, and may be attenuated by lesions of the ventral noradrenergic bundle [11]. Conversely, eating may be suppressed by administering a2-adrenoceptor antagonists, such as yohimbine [17,18].

Together, this evidence suggests the relevant role of the noradrenergic system in the regulation of feeding behavior, indicating the possible specific actions of endogenous and exogenous NE in influencing human nutrition.

### 1.3. The Noradrenergic System and Human Metabolism

Besides its relevant direct, hypothalamus-based actions on feeding regulation, the noradrenergic system is indirectly implied in various endocrine networks controlling humane nutrition. Namely, ghrelin, neuropeptide Y (NPY), cholecystokinin (CCK), leptin, oxytocin, and insulin, six hormones involved in the regulation of feeding and satiety, may interact with noradrenergic peripheral networks [19]. Peripheral ghrelin signaling, transmitted along the vagus nerve to the nucleus tractus solitarius (NTS), enhances NE in the arcuate nucleus of the hypothalamus, thus stimulating feeding through a1- and b2-noradrenergic receptors [20]. The vagus nerve and NTS have been also implied in the control of satiety, since lesions of these structures abolish the effect of CCK, a satiety hormone activating adrenergic/noradrenergic NTS neurons, suggesting that adrenaline and NE may work as anorectic signals at the NTS [21]. The sympathetic system, moreover, can inhibit leptin secretion, with a specific action of adrenergic regulation on decreasing serum leptin and insulin levels during exercise in patients with AN and BN [22]. Leptin and CCK, moreover, are involved in the regulation of oxytocin secretion and hypothalamic release of noradrenaline, since Kutlu and colleagues demonstrated that leptin may inhibit oxytocin secretion by lowering NE neurotransmission in the PVN, thus possibly modulating feeding behavior [23]. Lastly, adrenergic activity, together with cortisol, may regulate the NPY adipogenic system, thus influencing central feedbacks of adiposity [24]. The influence of cortisol in regulating the NE control of feeding behavior, nonetheless, extends to different metabolic systems. The hypothalamic–pituitary–adrenal axis may be overly active in patients with altered eating behavior, causing sustained high cortisol levels [25,26,27,28].

### 1.4. The Catecholaminergic System and Eating Disorders

Eating Disorders (EDs) are psychiatric disorders characterized by persistent disturbances of eating behaviors, resulting in the altered consumption or absorption of food and significantly impair physical health or psychosocial functioning [29]. EDs are associated with relevant personal and societal burden, including high mortality and significant medical and psychiatric comorbidity [30].

Current research suggests that multiple pathogenetic factors are involved in the development of EDs. Genetic and neurodevelopmental factors have been hypothesized to explain the significant comorbidities between EDs and a series of psychiatric disorders, such as Anxiety Disorders, Mood Disorders, and Autism Spectrum Disorder [31,32]. Additionally, environmental and cultural factors have frequently been involved in the pathogenesis of EDs [30].

The role of the catecholaminergic systems in the pathogenesis of EDs has been addressed by a series of studies; in particular, a relevant systematic review by Kontis and Theochari assessed the involvement of dopamine in the development and management of Anorexia Nervosa (AN) [33]. The authors documented and classified specific studies in the field of preclinical research, genetics, neuroimaging, biochemistry and laboratory medicine, psychiatry and pharmacology, documenting major alterations in the transmission of DA in individuals with AN. The review, moreover, described and classified the most relevant drug treatments acting on the dopaminergic system and involved in the management of EDs, thus indicating the essential need for further research to develop targeted treatments for these conditions [33]. Theoretical papers, moreover, have specifically assessed the contribution of NE system in the development of EDs. Nunn and colleagues [34] proposed that genetically determined NE dysregulation, interacting with epigenetic factors, may lead to insula dysfunction and homuncular representation alteration in individuals with AN [34]. 

### 1.5. Aims of the Present Review

Despite the evidence on the links between catecholaminergic transmission and feeding behavior, no specific review has systematically addressed the relationship between the noradrenergic system and EDs.

This systematic review aims to highlight the existing literature on the role of the noradrenergic system in the pathogenesis, transmission and treatment of EDs. 

The research questions addressed include: (1) Is there documented evidence of involvement of the noradrenergic system in the pathogenesis of EDs? (2) Is there a documented theorical background for the development and testing of treatments for EDs acting on the noradrenergic system?

## 2. Methods

This systematic review was performed in August 2021 by browsing the following databases: Medline (PubMed), Cochrane Library, Clinicaltrials.gov. Research was enhanced by searching the most relevant websites of guidelines and clearinghouses and in the most important etextbooks sites.

The Preferred Reporting Items for Systematic Reviews and Meta-Analyses guidelines (PRISMA) flowchart [35] was used to determine the transparent exclusion of published literature with defined reasons. Pertinent selected works were tabulated with regards to study design, number and type of participants, type of study, main results. Study types were classified according to the framework used in a recent systematic review on the role of dopaminergic system in AN [33]: preclinical; clinical studies addressing cerebrospinal, plasma and urinary concentrations; neuroimaging; genetics; treatment studies.

Inclusion criteria: Date: published between January 2000 and July 2021. Population: humans or non-human mammals; Language: English; Study Design: RCT, cohort studies, cross-sectional studies, retrospective studies, case reports; Outcomes: reported.

Exclusion: Population: other than humans or non-human mammals; Language: other than English; Study design: descriptive studies, reviews, protocols; Outcomes: unreported.

Search string: ((noradren* OR norepinephrine[MH]) AND (eating disord* OR bulimia nervosa OR binge-eating disorder OR anorexia nervosa OR avoidant/restrictive food intake disorder)).

A total of 341 original articles were identified with the initial search and checked by two independent investigators (J.P. and D.M.C.), and disagreements among reviewers were resolved through a mediator (M.L.). Thirty-five studies were included in the final analysis after 306 studies were excluded on the basis of exclusion/inclusion criteria. 

**Figure 2:**
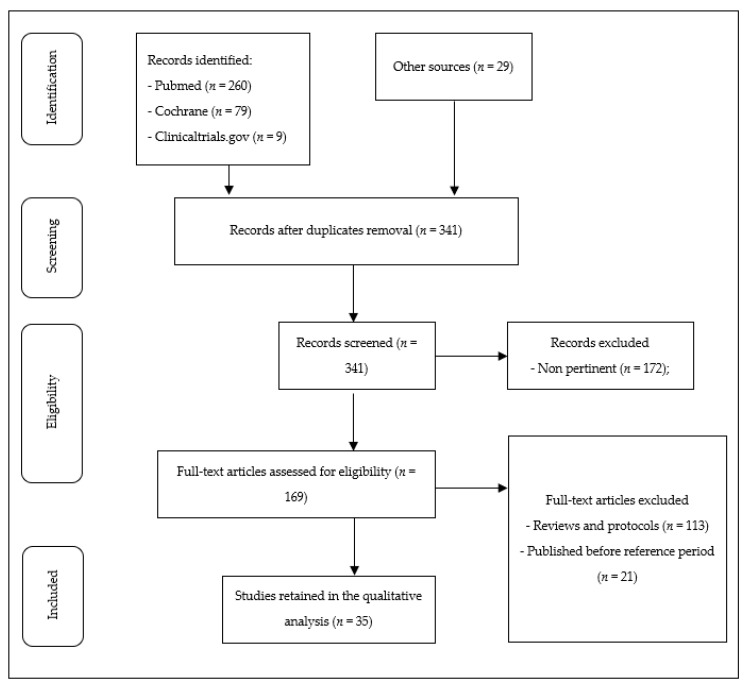
Flowchart of the study.

The flow chart of the study is reported in Figure 2.

## 3. Results

### 3.1. Studies Characteristics

A detailed description of the selected articles is reported in Table 1.

### 3.2. Preclinical Studies

Along with preclinical and clinical research unveiling the link between noradrenergic regulation and feeding behavior, a series of studies have investigated the role of noradrenergic dysregulation in the pathogenesis of EDs.

The effect of the serotonin and noradrenaline reuptake inhibitor (SNRI) sibutramine, an anti-obesity treatment, was studied in 10 rats by Tallet and colleagues [36]. The authors found that sibutramine dose-dependently reduced food intake, time spent feeding, and increased the frequency of resting, concluding that the anorectic efficacy of this drug is due to an acceleration in behavioral satiety.

Bello and colleagues [37] measured the neural activation by c-Fos reactivity to food in bingeing and non-bingeing rats under stress. The authors found an increased activation in the dorsal medial prefrontal cortex (mPFC) in stressed animals previously exposed to the binge-eating but administering selective alpha-2A adrenergic agonist guanfacine reduce binge-like eating. The authors hypothesize that mPFC is differentially activated in response to stress under different dietary conditions.

In a further study, the same group [38] tried to investigate the influence of dietary-induced binge eating on the neuronal activity of the LC-norepinephrine system. Rats were intermittently exposed to sweetened fat, with or without intermittent calorie restriction, and compared to control rats. The experimental group showed that binge rats presented significantly reduced LC discharge rates compared with control, supporting the idea that dietary-induced binge eating modifies the neural response of LC neurons.

Romano and colleagues [39] assessed the anti-binge effect of oleoylethanolamide (OEA), a lipid-derived messenger involving central noradrenergic and oxytocinergic neurons, in a rat model of binge-like eating. Systemically administered OEA dose-dependently prevented binge-eating. Relevantly, this effect was linked with decreased activation of brain areas responding to stress, and to stimulation of areas involved in the control of food intake, such as the ventral tegmental area (VTA) and the PVN. Concurrently, OEA modulated monoamine transmission in key brain areas involved in homeostatic and hedonic control of feeding, suggesting that OEA might represent a pharmacological target for the treatment of binge-like eating behavior.

Recently, Hicks and colleagues [40] examined the role of the noradrenergic system in binge-like eating, administering the alpha-1 adrenergic receptor antagonist prazosin to food-restricted rats. Prazosin reduced palatable responses, suggesting that this treatment preferentially increased the motivational properties of the palatable diet.

### 3.3. Genetic Studies

Genetic studies have documented possible contributions of polymorphisms in NE transporters in the pathogenesis of ED.

Urwin and colleagues [41] hypothesized an involvement of the noradrenaline transporter gene (NET) in the genetic transmission of AN. The authors performed a PCR-amplification of an AAGG repeat island in the NET gene promoter, revealing a novel sequence named the NET gene promoter polymorphic region (NETpPR). A 4-bp deletion (S4) or insertion (L4) in this sequence resulted in the net loss or gain, respectively, of a putative Elk-1 transcription factor site. Then, performing transmission disequilibrium tests (TDT) with 87 Australian groups (patient plus biological parents), the authors demonstrated a preferential transmission of L4 from parent to patients with ANR. This data lead to the hypothesis that L4 or a DNA variant in linkage disequilibrium may double the genetic risk of developing ANR.

These results were further examined by a second study from the same group [42]. The authors conducted association study with a functional polymorphism (MAOA-uVNTR) in the promoter of the coding gene for monoamine oxidase A (MAOA), an enzyme deputed to metabolize NE. A transmission disequilibrium test performed on 95 families of ANR females and their biological parents showed the main effect of the longer, more transcriptionally active form of the MAOA-uVNTR (MAOA-L) to be statistically non-significant. Then, the authors stratified the MAOA-uVNTR TDT data according to the NETpPR genotype of the patients, and NETpPR allele transmitted from NETpPR-S4/L4 heterozygous mothers. The analyses revealed that receiving an MAOA-L allele more than doubles the genetic risk to develop ANR, when an individual also carries a NETpPR-L4 homozygosity.

Relevantly, Hu and colleagues [43] tried to replicate the association documented by Urwin and colleagues [41] concerning the NETpPR polymorphism and the transmission of AN in a wider sample. The authors analyzed the NETpPR in 142 family trios, consisting of 67 patients with ANR, 48 with ANBP and 27 unclassified AN. This research documented no significant transmission distortion for any of the analyzed alleles, failing to replicate the association found by Urwin and colleagues. The authors concluded that the initial finding was a false-positive association, or that these different results should be attributed to difficulties replicating a gene with a small effect.

### 3.4. Clinical Studies Assessing Concentrations in Body Tissues and Fluids

In addition to these studies, a series of authors have investigated the presence and modifications of noradrenaline and its metabolites in the plasma and urine. The most relevant metabolite of noradrenaline in primates is 3-methoxy-4-hydroxyphenylglycol (MHPG), and MHPG in brain tissue, cerebrospinal fluid, plasma, and urine reflects the turnover of noradrenaline [70].

In a large study on the prevalence of migraine and the tyrosine metabolism in patients with EDs, D’Andrea and colleagues [44] measured the plasma levels of NE, DA and elusive amines in young AN and BN individuals and HC. The study showed that levels of NE were lower in the ED patients with respect to HC, suggesting that abnormalities of NE circuitries may play a role in the pathogenesis of EDs.

Plasma levels of NE, together with a series of other neurotransmitters and measures of autonomic system activity, were assessed in a group of patients with AN and HC by Lechin and colleagues [45]. Variables were measured at rest, during orthostasis, and after five minutes of exercise. The authors documented that patients with AN had adrenal sympathetic overactivity and neural sympathetic underactivity, as shown by a predominance of circulating adrenaline over NE levels, both at rest, during orthostasis, and after exercise. 

The same group [46] investigated variations in blood pressure, heart rate, and circulating neurotransmitters, (NE, adrenaline, DA, platelet serotonin - 5-HT, free plasma 5-HT) during rest, orthostasis and exercise, after treatment with amantadine, a drug which abrogates adrenal sympathetic activity by acting at the C1(Ad) medullary nuclei responsible for this branch of the peripheral sympathetic activity. The study was conducted on a sample of 22 females with AN. The authors found that amantadine abolished symptoms of AN and normalized autonomic and cardiovascular parameters. Abrupt and sustained increases in the serum noradrenaline:adrenaline ratio and disappearance of abnormal plasma glucose elevation were documented, leading to the hypothesis that AN depends on hypomotility of the gastrointestinal tract plus hyperglycemia, both of which are triggered by adrenal sympathetic hyperactivity.

The activity of NE and the autonomic nervous system was also studied by Yoshida and colleagues [47]. Nine patients with AN were assessed for weight, RR interval (RRI), heart rate variability, endocrine function, and energy expenditure before and after the start of refeeding. After short-term refeeding, the mean heart rate increased from 54.9 to 69.4 beats per minute. Relevantly, NE tended to increase, together with urine C-peptide, insulin-like growth factor-1 (IGF-1), and fT3.

Brambilla and colleagues [48] assessed the effects of cognitive-behavioral therapy (CBT) on central dopamine, NE, and 5-HT secretion in a group of 50 female inpatients with AN and BN. To do so, the authors studied possible variations of specific dopamine (blood homovanillic acid, HVA) and NE (MHPG) metabolites, as well as the 5-HT transporter. CBT significantly improved the psychophysical aspects of the considered diseases, but metabolites of dopamine and noradrenaline were not significantly different, despite variations in 5-HT transporter being documented in patients with BN. These results suggest a possible action of CBT in BN through changes in the serotoninergic system’s function.

In a relevant study by Bartak and colleagues [49], an in vivo microdialysis technique was used for the assessment of NE, dihydroxyphenylalanine, dihydroxyphenylacetic acid, and glycerol concentrations in subcutaneous adipose tissue of 10 individuals with AN and 10 healthy controls (HCs), both undergoing an exercise test. Basal NE concentrations resulted increased in the AN patients in comparison with the controls, and during exercise a local increase in the concentration of NE in the AN patients only in the adipose tissue. Interestingly, basal and exercise-induced NE plasma levels did not differ between the AN and HC groups. The authors concluded that there is evidence of elevated baseline and exercise-induced sympathetic nervous activity and exercise-induced lipolysis in the adipose tissue of individuals AN.

In another microdialysis study on adipose tissue NE concentration, Nedvikova and colleagues [50] investigated five AN patients and six HC under basal conditions and after the local administration of maprotiline, an inhibitor of NE re-uptake. Basal adipose NE levels were significantly increased in AN patients compared to the controls (106.0+/−20.9 vs. 40.0+/−5.0 pg/mL). The local administration of maprotiline induced a significant increase in adipose NE levels (AN patients: 440.0+/−28.6 vs. 202.0+/−33.0 pg/mL in the HCs) in both groups. These results suggested that markedly increased subcutaneous abdominal adipose tissue NE levels in AN patients compared to HCs reflect increased sympathetic nervous system activity, but not altered membrane noradrenergic transporter system in AN patients.

Dostalova and colleagues [22] investigated the effects of cycle ergometer exercise on plasma NE, leptin, glycerol, and insulin levels in 10 patients with AN and in 15 HCs. Basal and exercise-induced plasma NE and glycerol levels did not differ significantly between the groups, while differences were found for leptin and insulin. These results suggested that NE was not responsible for the different response of leptin to exercise in AN.

Vaz-Leal and colleagues [51] analyzed the capability of a set of neurobiological and psychopathological variables to discriminate BN from HC. Participants were compared for psychopathology and neurobiological parameters reflecting hypothalamic–pituitary–adrenal axis activity (morning cortisol before and after dexamethasone) and monoamine activity (24-h urinary excretion of NE, serotonin, dopamine, and their main metabolites: MHPG, 5-hydroxyindoleacetic acid, and HVA). BN patients had higher psychopathological scores, and lower excretion of serotonin and dopamine than controls, as well as lower ability to suppress cortisol. Nonetheless, NE and its metabolites were found to not be significantly different between BN and HCs, and no significant correlation with psychopathological measures was found.

Zheng and Yang [52] administered mirtazapine to 100 patients with AN and dyspepsia, additionally treating with metformin one half of the sample. The authors found gastric juice pH increased after treatment in both groups, as well as body weight, serum NE, 5-HT and DA levels, showing NE plasma levels may be influenced by metformin combined with mirtazapine.

Rigaud and colleagues [53] investigated the thermic effect of food (TEF) in relation to subjective feelings and serum hormone levels in a group of 15 individuals with AN and 15 HCs after three gastric loads (0, 300, 700 kcal) infused by a nasogastric tube. In AN, the loads induced an increase in TEF higher than that in HCs. Only in AN, a load-dependent decline in the basal level of beta-endorphin, an increase in plasma ACTH, and an increase in cortisol, NE, and DA levels were noted. The authors concluded that in AN women, blindly infused loads induced a dose-dependent increase in TEF, correlating with an increase in serum cortisol, ACTH, and catecholamines.

### 3.5. Brain Imaging Studies

No brain imaging study meeting the criteria of this review was identified investigating the noradrenergic system in patients with ED.

### 3.6. Pharmacological Studies

Relevant research has provided a theoretical framework for treating these patients with NE-based drugs. The studies of Kaye and colleagues [71] have directly documented the key role of the noradrenergic system in influencing vulnerability to EDs. The authors found that bingeing and vomiting activates the sympathetic nervous system in individuals with BN, increasing in the duration and the peak increase of plasma NE, while prolonged abstinence from these behaviors reduced basal plasma and CSF NE levels.

As for the studies included in this systematic review, a series of pharmacologic treatments influencing the NE system have been found to have an effect of disrupted food regulation and symptoms of EDs. Thus, studying the mechanism of action of these drugs may contribute to understanding the role of NE in the pathogenesis of EDs.

Milano and colleagues [54] studied the effect of sibutramine, a SNRI, in the treatment of obese patients with BED in a randomized, double-blind, placebo-controlled study. Binge frequency was the primary outcome measure and resulted significantly lower in patients receiving sibutramine than that of those given placebo.

Venlafaxine, a SNRI acting as a weak NE reuptake inhibitor at low doses (75 mg/day) [72] indicated in the treatment of depressive disorders, has been studied by Lanzarone and colleagues [55] in a controlled trial on 30 patients with BED. The patients were treated with CBT+/−paroxetine or venlafaxine and assessed for binge frequency and psychopathology. The data showed that CBT alone induced a greater reduction in depression and hypomania whereas pharmacological treatment appeared to control primarily the impulsiveness of food intake.

Duloxetine, a SNRI indicated in the treatment of depressive and anxiety disorders, as well as chronic pain, has been investigated by a series of articles in the field of EDs; Bernardi and Pallanti [56] reported the successful treatment of a case of refractory binge eating disorder (BED) with duloxetine, resulting in complete remission of the patient’s bingeing behaviors, demonstrating that inhibition of 5-HT and NE reuptake by duloxetine significantly reduced food intake, suggesting that this may be a novel approach for the treatment of obesity.

Leombruni and colleagues [57] assessed the efficacy of duloxetine in 45 obese patients with binge eating. The authors documented a significant reduction in binge frequency, weight, and psychopathology, suggesting that duloxetine may be a successful option to reduce binge eating and depressive symptoms in obese patients with binge eating.

Guerdjikova and colleagues [58] investigated the effect on binge frequency of duloxetine in the treatment of 40 patients with BED and depression in a double-blind, placebo-controlled trial. Duloxetine was superior to placebo in reducing binge frequency, weight, and psychopathology.

Similar evidence has been documented for milnacipran, a dual-acting antidepressant that blocks both serotonin and noradrenaline reuptake., in the treatment of 16 outpatients with BN by El-Giamal and colleagues [59], showing a reduction in weekly binge eating and vomiting frequency from baseline to the end of treatment; 3 patients completely stopped binge eating and purging. Psychopathology improved as well, suggesting that milnacipran may be promising in the treatment of BN.

Further data are available on the effects of reboxetine, a selective norepinephrine reuptake inhibitor (NRI), in the treatment of ED. A case report published by Willeit and colleagues [60] documented a successful treatment of BN and resistant depression with NRI reboxetine, producing initial evidence for the effect of drugs selectively targeting the NE system in the management of ED.

In the same year, El-Giamal and colleagues [61] documented the use of reboxetine for seven outpatients with BN, showing reduced binge and purging frequency, together with a reduction in depressive psychopathology.

Similarly, Fassino and colleagues [62] conducted an open label clinical trial, administering reboxetine to 28 outpatients with BN. The authors documented that 60% of the patients were responsive to treatment (50% decrease of BN behaviors), with a reduction in psychopathological scores for ED and depression, suggesting a favorable effect of reboxetine in the treatment of symptoms and psychopathology of BN.

The effect of reboxetine has been studied by Silveira and collaborators [63] on the treatment of BED. Nine outpatients with BED and obesity were treated with reboxetine, and the binge frequency represented the primary outcome measure, while weight, body mass index (BMI) and psychopathology represented the secondary outcomes. After treatment with reboxetine, a significant reduction in binge frequency and psychopathology was documented, leading to the conclusion that reboxetine may be an effective agent in the treatment of obese individuals with BED.

Relevant data may be found in studies assessing the effect of dasotraline, a novel serotonin-norepinephrine-dopamine reuptake inhibitor (SNDRI), in the treatment of EDs.

Mattingly and colleagues [64] investigated the effect of dasotraline in the treatment of 533 patients with BED, documenting dasotraline to be safe and well tolerated. In a series of studies conducted on a similar sample, the same research group documented the efficacy of dasotraline in the treatment of BED, showing positive effects on binge frequency, weight, and psychopathology [65,66,67].

Atomoxetine, a highly selective norepinephrine reuptake inhibitor associated with weight loss, was studied in a randomized, double-blind, placebo-controlled, flexible dose trial, assessing reduction in binge frequency in 40 patients with BED. Compared with placebo, atomoxetine was associated with a greater reduction in binge frequency, BMI, and psychopathology, showing efficacy in the treatment of BED [68].

Relevantly, desipramine, an antidepressant medication inhibiting the reuptake of noradrenaline and, to a lesser extent, serotonin, appears to reduce binge eating and improve comorbidities in short-term treatments [73,74]. The effect of this drug has been documented in one study meeting the criteria of this systematic review. The authors found treatment with desipramine was associated with clinical response and remission in a subgroup of patients with BN, while non-responders could be identified at in the first two weeks of treatment [69].

## 4. Discussion

This systematic review assessed the role of the noradrenergic system in the pathogenesis and treatment of EDs. A total of 35 studies were included, of whom 5 were preclinical trials on rats, and 29 were clinical studies on humans.

The human nutrition encompasses a complex series of biological, genetic, and cultural factors [75,76]. EDs represent an increasing issue in the field of nutrition and mental health, involving a series of psychiatric, metabolic, nutritional, and pharmacologic challenges for clinicians. The etiology of these conditions is now strongly considered multifactorial, with relevant contributions from genetic, epigenetic, neurobiological, psychopathologic, and endocrine factors [29]. A series of evidence addresses the relevant role of catecholamines, with specific reference to NE, in the pathogenesis of EDs.

### 4.1. Preclinical Studies

Preclinical studies have agreed in documenting a significant role of the noradrenergic system in the pathogenesis of EDs under experimental conditions. Relevantly, binge eating has been found to activate the mPFC in stressed rats, an effect that could be reduced by alpha-2A adrenergic agonist guanfacine [37], while dietary-induced binge eating may alter the neural response of LC neurons, via reducing their discharge rates [38]. Administering the anti-binge eating lipid molecule OEA according to this framework has been shown to decrease activation of brain areas responding to stress, and enhance the activity of the VTA and the PVN, brain areas involved in the control of food intake [39]. A direct, anti-binge eating behavioral effect has been documented for to molecules modulating the noradrenergic transmission in the CNS, namely sibutramine (a SNRI) and prazosin (an alpha-1 adrenergic receptor antagonist modulating addiction-like behaviors) [36,40]. Together, this evidence indicates an involvement of the noradrenergic brain system in the development of binge-like behaviors, frequently mediated by the altered activation of brain areas deputed to the control of stresses, and a role for drugs modulating the noradrenergic system to influence binge eating in preclinical settings. No evidence has been found in recent literature concerning restricting, purging, and anorectic-like behaviors.

### 4.2. Genetic Studies

Genetic studies included in this review have reported conflicting evidence. The research by Urwin and colleagues documented the hypothesis that receiving specific NET and MAOA variants and may increase the genetic risk to develop ANR [41,42]. Nonetheless, a more recent study on a greater sample failed to replicate these findings [43]. Thus, scientific evidence available so far does not indicate unanimous evidence for the existence of a possible hereditary, noradrenergic-mediated risk in the development of AN. Notably, no evidence has been found accounting for possible genetic contributions in the pathogenesis of BED. Nonetheless, a recent systematic review has documented a relevant role of a dopaminergic-mediated, genetic influence on the development of BED [77], thus future studies may expand current knowledge in this field.

### 4.3. Clinical Studies Assessing Concentrations in Body Tissues and Fluids

Clinical studies assessing variations of NE in bodily fluids and tissues have produced conflicting evidence. Plasma NE has been found to be reduced at baseline in AN and BN in a series of studies, both as absolute levels [44] and when compared to circulating adrenaline [45], while a series of other studies [22,49,51] did not find a difference in basal plasma NE levels between patients with AN/BN and controls. As for the studies investigating possible modifications in NE levels of AN/BN patients after exercise, no difference between these individuals and HC has been documented [22,49,51]. Concerning variations of catecholamine levels due to therapeutical interventions, NE has been shown to increase after refeeding [47], but not after CBT [48]. Together, these results do not indicate a direct evidence of specific baseline, exercise-induced, or treatment-induced alterations in the simple measurement of NE plasma levels in patients with AN or BN, with respect to HC. It is possible that future studies investigating variations of this neurotransmitter or its metabolites in the brain or cerebrospinal fluid (CSF) under experimental conditions may reveal more specific elements differentiating patients with ED from HC. No studies were available concerning BED patients. 

### 4.4. Brain Imaging Studies

No brain imaging (magnetic resonance or positron emission tomography) study was found investigating the noradrenergic system in patients with EDs. Since a series of studies on this field exist on the roles of dopamine, as documented by a recent systematic review [78], future research should address this gap in the literature.

### 4.5. Pharmacological Studies

A number of pharmacological studies have documented the effect of drugs influencing the noradrenergic transmission in the CNS on the management of different EDs. SNRIs represent the most frequently studied molecules, with specific research targeting the effects of sibutramine, venlafaxine, duloxetine and milnacipran. SNRIs have been documented to have a positive effect in reducing binge frequency and psychopathology in BED [54,55,56,57,58] and, in the case of milnacipran, the frequency of binge and purging, as well as depressive symptoms in BN [59]. A relevant effect has been documented for the NRI reboxetine as well, since this drug may reduce improve and purge frequency and psychopathology in BN [60,61,62] and BED [63]. A positive effect in the treatment of BED has been documented for highly selective NRI atomoxetine as well, with improvement in binge frequency, weight, and psychopathology. Besides SNRIs and NRIs, a series of trials from one study group have investigated the effects of dasotraline, a serotonin-norepinephrine-dopamine reuptake inhibitor (SNDRI) in the treatment of BED, reducing binge frequency, weight and psychopathology. A positive effect of desipramine, a drug inhibiting the reuptake of norepinephrine and serotonin in the presynaptic neuronal membrane in reducing the frequency of binge and purging in individuals with BN, has been documented as well [69].

Taken together, these results indicate that psychopharmacologic therapies modulating the noradrenergic activity, namely SNRI, NRI, and SNDRI, may serve as effective treatments for binge and purge frequency, weight alterations, and psychopathology for BN and BED. No recent data concerning the use of these drugs in patients with AN was found. Research in this field is urgently needed, since scientific evidence exists supporting only a few molecules in the treatment of AN [79,80,81].

### 4.6. Limitations of the Study

This study has some limitations. A major point to be considered when assessing the biochemical dysregulation in psychiatric disorders is the difficulty to clearly determine whether the documented modifications represent a causal factor or consequence of the disease. This can be particularly challenging when considering the field of EDs, since malnutrition and the metabolic imbalance determined by the underlying disorder may potentially alter endocrine and neuropsychological systems in the body [29]. Södersten and colleagues [82] specifically addressed this issue, considering the role of dopaminergic modifications in the pathogenesis of AN. The study concluded that the altered dopaminergic action found in individuals with AN are more likely to represent normal responses to starvation than primary signs of the disease. To confirm this thesis, the authors reported the evidence that effective treatments for AN more frequently attempt to normalize disordered eating behavior, instead of directly treating the mental symptoms emerging from malnutrition. Some of these considerations may be retained as valid in the light of the present systematic review, since NE alterations documented in individuals with AN may respond to nutritional interventions [53]. Nonetheless, genetic studies documenting a directly NE-mediated, genetically inherited risk of developing AN seem to challenge this hypothesis [41,42]. Thus, further research, particularly in the field of molecular genetics, is required to directly address this compelling issue.

As a second limitation, although we aimed at defining the evidence of involvement of the noradrenergic system in the pathogenesis of EDs, different studies did not concern all clinical entities of EDs. Namely, genetic studies were related to ANR only, as well as no clinical study assessing body concentrations of NE was found. Relevantly, no study was identified concerning preclinical or clinical aspects of the relationship between the noradrenergic system and the avoidant/restrictive food intake disorder. Thus, a further thorough systematic review in this field should include new research assessing both preclinical and clinical variables in these less studied conditions.

## 5. Conclusions

The present systematic review identified a series of key data on the relationship between the noradrenergic system and EDs. EDs represent a group of complex conditions with a multifactorial pathogenesis, and no specific review has systematically addressed the relationship between the noradrenergic system and EDs so far. According to our results, preclinical studies in rats demonstrated the involvement of the noradrenergic brain system in binge-like behaviors, with a key mediating role played by the activation of stress circuits. Genetic studies documented conflicting evidence in the possibility of a noradrenergic-mediated, genetically transmitted increased risk to develop AN. Clinical studies on body fluid concentrations reported non-unanimous evidence for the existence of absolute alterations in plasma NE values in patients with AN and BN. Pharmacological studies documented an efficacy of SNRIs, NRIs, and SNDRIs in the treatment of BN and BED. Still, insufficient evidence is available concerning the noradrenergic-mediate heritability of BED and BN, and psychopharmacological treatments targeting the noradrenergic system in AN. Further in vivo experimental studies and psychopharmacological randomized controlled trials are urgently needed to provide solid scientific evidence in the treatment of these expanding and increasing psychiatric and metabolic disorders. 

## Figures and Tables

**Table 1:** Characteristics and main results of the included studies.

Study	Subjects	Methods of the Study/Administered Interventions	Outcomes	Main Results
Preclinical studies				
Tallet et al., 2009 [36]	Non-deprived rats (*n* = 10)	Sibutramine	Food intake, satiety, weight gain	Sibutramine reduced food intake, time spent feeding and increased resting
Bello et al., 2019 [37]	Binge (*n* = 20), restrict and binge (*n* = 27), naive (*n* = 22) rats	Exposure to highly sweetened fat with/without caloric restriction	Neuronal activity of the locus coeruleus NE system	Restrict-Binge and Binge showed reduced LC discharge rates compared with naive
Bello et al., 2020 [38]	Restrict Binge (*n* = 12), Binge (*n* = 12), Restrict (*n* = 12), Naive (*n* = 12) rats	Guanfacine	Binge frequency, stress response, plasma catecholamines, and body composition	In the binge group, guanfacine increased calories consumed and increased binge food consumption
Romano et al., 2020 [39]	N = 144, different subgroups with induced binge-eating rats	Oleoylethanolamide	Binge frequency, c-fos expression, activation of specific brain areas	Oleoylethanolamide reduced binge frequency and increased hypothalamic NE tone
Hicks et al., 2020 [40]	Binge (*n* = 39 rats)	Prazosin	Responses for palatable food	Prazosin preferentially increased the motivational properties of the palatable diet
Genetic studies				
Urwin et al., 2002 [41]	ANR + biological parents (*n* = 87 trios)	PCR-amplification of an AAGG repeat island in the NE transporter gene promoter region	Analysis of transmission disequilibrium	Preferential transmission of a 4-bp deletion, resulting in net gain of a putative Elk-1 transcription factor site from parent to child with ANR
Urwin et al., 2003 [42]	ANR + biological parents (*n* = 95 trios/duos)	Association study	Association study with a functional polymorphism (MAOA-uVNTR) in the promoter of MAOA gene	Receiving an MAOA-L allele more than doubles the risk for developing ANR, conditional on an individual also being a NETpPR-L4 homozygote
Hu et al., 2007 [43]	ANR + biological parents (*n* = 67), ANBP (*n* = 48), unclassified AN (*n* = 27)	Patients genotyped for the NETpPR polymorphism	Analysis of transmission disequilibrium	No significant transmission distortion for any of the alleles we detected with the putative L4 risk transmitted
Clinical studies addressing concentrations of NE in body tissues/fluids				
D’Andrea et al., 2009 [44]	AN (*n* = 89), BN (*n* = 36), HC (*n* = 27)	Measurement of NE plasma levels	NE plasma levels	Levels of NE were lower in the ED patients with respect to the control subject.
Lechin et al., 2010 [45]	AN (*n* = 22), HC (*n* = 22)	BP, HR, and plasmatic neurotransmitters	Measurements during resting, orthostasis, exercise	AN individuals showed increased adrenaline:noradrenaline levels, across all tested conditions
Lechin et al., 2011 [46]	AN (*n* = 22)	Amantadine	BP, HR, and circulating neurotransmitters	Amantadine abolishes symptoms of AN, normalizes autonomic parameters, increases noradrenaline:adrenaline ratio
Yoshida et al., 2006 [47]	AN (*n* = 9)	Refeeding	HR, endocrine measurements including NE	Refeeding increased HR and NE
Brambilla et al., 2010 [48]	ANR (*n* = 14), ANBP (*n* = 14), BN (N = 22).	CBT	MHPG, psychopathology	CBT improved psychopathology, but no changes in MHPG were registered.
Bartak et al., 2004 [49]	AN (*n* = 10), HC (*n* = 10)	In vivo microdialysis in subcutaneous adipose tissue	Basal and exercise-stimulated plasma DNA subcutaneous adipose NE	Basal and exercise stimulated NE in adipose tissue was more increased in AN
Nedvikova et al., 2005 [50]	AN (*n* = 5), HC (*n* = 6)	In vivo microdialysis in subcutaneous adipose tissue, before and after maprotiline	Subcutaneous adipose NE levels	Basal NE levels were significantly increased in AN patients compared to the controls, while post-maprotiline levels increased in both groups.
Dostalova et al., 2007 [22]	AN (*n* = 10), HC (*n* = 15)	A 45-min cycle ergometer exercise	Basal and exercise induced plasma endocrine measures including NE	Basal and exercise induced NE did not change in both groups
Vaz-Leal et al., 2011 [51]	BN (*n* = 75), HC (*n* = 30)	Comparison between BN and HC, analysis of relationship between neuroendocrine and psychopathological measures	Psychopathology and parameters reflecting hypothalamic-pituitary-adrenal axis activity and monoamine activity (24-h urinary excretion of NE, serotonin, dopamine, MHPG, 5-HIAA, HVA).	NE and its metabolites were found not significantly different between BN and HC, and no significant correlation with psychopathological measures was found.
Zheng and Yang, 2014 [52]	AN (*n* = 100)	Half of the patients received mirtazapine, while the other half received mirtazapine + metformin	Gastric juice pH, weight, HAMD, HAMA, NE, 5-HT, dopamine and blood glucose levels were compared	After treatment, gastric pH and weight increased. HAMA and HAMD scores decreased, while plasma NE, 5-HT and DA increased in both groups.
Rigaud et al., 2007 [53]	AN (*n* = 15), HC (*n* = 15)	Three gastric loads infused by a nasogastric tube.	Thermic effect of food, feelings, plasma release of a series of hormones and neurotransmitters	Only in AN, a load-dependent increase in norepinephrine, and dopamine levels after the 700-kcal load only (*p* < 0.05) were noted.
Pharmacological studies				
Milano et al., 2005 [54]	BED (*n* = 20)	Sibutramine	Binge frequency	Sibutramine more effective than placebo in reducing binge frequency.
Lanzarone et al., 2014 [55]	BED (*n* = 30)	CBT vs CBT + paroxetine/venlafaxine	Binge behavior, impulse regulation, eating behavior, psychotic conditions	CBT showed greater improvement in depression, hypomania, and control eating behavior; whereas pharmacological treatment appears to improve impulsiveness of food intake.
Bernardi and Pallanti, 2010 [56]	BED (*n* = 1)	Duloxetine	Binge frequency	Binge behavior completely remitted after duloxetine
Leombruni et al., 2009 [57]	BED or sub-threshold BED (*n* = 45)	Duloxetine	Binge frequency, BES, Beck depression inventory, BMI, CGI, EDI-3	All the outcome measures improved in the whole sample
Guerdjikova et al., 2012 [58]	BED (*n* = 40)	Duloxetine	Binge and purging frequency, depressive ratings	Duloxetine was superior to placebo in reducing binge frequency, CGI of illness and depression. Changes in BMI and measures of eating pathology, depression, and anxiety did not differ between the two groups.
El-Giamal et al., 2003 [59]	BN (*n* = 16)	Milnacipran	Binge and purging frequency, depression ratings	From baseline to the end of treatment, patients treated with milnacipran showed a significant reduction in binge and vomiting frequency and depressive ratings.
Willeit et al., 2000 [60]	BN (*n* = 1)	Reboxetine	Binge and purging frequency, depressive ratings	After introduction of reboxetine, the patient experienced a remission of BN and depressive symptomatology
El-Giamal et al., 2000 [61]	BN (*n* = 7)	Reboxetine	Binge and purging frequency, depression ratings, EDI, EDQ	Patients treated with reboxetine showed a reduction in binge and purging frequency and depressive ratings
Fassino et al., 2004 [62]	BN (*n* = 28)	Reboxetine	Bulimic behaviors, Hamilton Rating Scale for Anxiety and for Depression, Global Assessment Functioning, EDI-2, and Body Shape Questionnaire.	In 60% of the patients, bulimic behaviors reduced, and depression, global functioning and body perception improved.
Silveira et al., 2005 [63]	BED (*n* = 9)	Reboxetine	BMI, binge frequency, BES, CGI, quality of life	Reboxetine induced complete remission of BED and improved all clinical outcomes in patients completing the study.
Mattingly et al., 2019 [64]	BED (*n* = 533)	Dasotraline	Adverse effects, weight, metabolic parameters, EKG, and measures assessing potential for drug withdrawal	A 12 months of treatment with dasotraline (4–8 mg/d) was found to be safe and well-tolerated by the majority of patients with BED
Loebel et al., 2019 [65]	BED (*n* = 317)	Dasotraline	Binge frequency, CGI, YBOCS-BE	Dasotraline, compared with placebo, was associated with greater reductions in binge frequency, and greater improvements in CGI and YBOCS-BE
McElroy et al., 2020 [66]	BED (*n* = 315)	Dasotraline	Binge frequency, CGI, YBOCS-BE	Dasotraline, compared with placebo, was associated with greater reduction in binge frequency, and greater improvements in CGI and YBOCS-BE
Grilo et al., 2020 [67]	BED (*n* = 324)	Dasotraline	Binge behavior, impulse regulation, eating behavior, psychotic conditions	Dasotraline 6 mg/d was associated with improvement binge frequency. Improvement vs. placebo was observed for dasotraline 6 and 4 mg/d, CGI and YBOCS-BE
McElroy et al., 2007 [68]	BED (*n* = 40)	Atomoxetine	Binge frequency, weight measures, CGI, YBOCS-BE	Atomoxetine, compared with placebo was associated with greater rate of reduction in binge frequency, weight measures, CGI, YBOCS-BE
Walsh et al., (2006) [69]	BN (*n* = 77)	Desipramine	Binge and vomiting frequency	Desipramine was associated with clinical response in 18/77 patients for binge frequency and 15/77 for vomiting; remission occurred in 10/77 for binge and 10/77 for vomiting. Non-responders could be identified in the first 2 weeks of treatment.

Abbreviations: AN: Anorexia Nervosa; ANBP: Anorexia Nervosa, binge-purging subtype; ANR: Anorexia Nervosa, restrictive subtype; BED: Binge Eating Disorder; BN: Bulimia Nervosa; BP: blood pressure; CBT: cognitive-behavioral treatment; CCK: cholecystokinin; CGI: clinical global impression; CNS: central nervous system; DA: dopamine; ED: Eating Disorders; HAM-A: Hamilton Anxiety Rating Scale; HAM-D: Hamilton Depression Rating Scale; HC: healthy controls; HR: heart rate; HVA: homovanillic acid; LC: locus coeruleus; MAOA: monoamine oxidase A; MHPG: 3-methoxy-4-hydroxyphenylglycol; mPFC: medial prefrontal cortex; NE: noradrenaline; NET: noradrenaline transporter; NPY: neuropeptide Y; NRI: noradrenaline reuptake inhibitor; NTS: nucleus tractus solitarius; OEA: oleoylethanolamide; PNS: peripheral nervous system; PRISMA: Preferred Reporting Items for Systematic Reviews and Meta-Analyses guidelines; PVN: paraventricular nucleus; SNDRI: serotonin-norepinephrine-dopamine reuptake inhibitor; SNRI: serotonin and noradrenaline reuptake inhibitor; TFE: thermic effect of food; VTA: ventral tegmental area; YBOCS-BE: Yale-Brown Obsessive Compulsive Scale modified for Binge Eating; 5-HIAA: 5-hydroxyindoleacetic acid; 5-HT: serotonin.

## Abbreviations

AN: Anorexia Nervosa; ANBP: Anorexia Nervosa, binge-purging subtype; ANR: Anorexia Nervosa, restrictive subtype; BED: Binge Eating Disorder; BN: Bulimia Nervosa; BP: blood pressure; CBT: cognitive-behavioral treatment; CCK: cholecystokinin; CGI: clinical global impression; CNS: central nervous system; DA: dopamine; ED: Eating Disorders; HAM-A: Hamilton Anxiety Rating Scale; HAM-D: Hamilton Depression Rating Scale; HC: healthy controls; HR: heart rate; HVA: homovanillic acid; LC: locus coeruleus; MAOA: monoamine oxidase A; MHPG: 3-methoxy-4-hydroxyphenylglycol; mPFC: medial prefrontal cortex; NE: noradrenaline; NET: noradrenaline transporter; NPY: neuropeptide Y; NRI: noradrenaline reuptake inhibitor; NTS: nucleus tractus solitarius; OEA: oleoylethanolamide; PNS: peripheral nervous system; PRISMA: Preferred Reporting Items for Systematic Reviews and Meta-Analyses guidelines; SNDRI: serotonin-norepinephrine-dopamine reuptake inhibitor; SNRI: serotonin and noradrenaline reuptake inhibitor; TFE: thermic effect of food; YBOCS-BE: Yale-Brown Obsessive Compulsive Scale modified for Binge Eating; 5-HIAA: 5-hydroxyindoleacetic acid; 5-HT: serotonin.
